# Predictors of Blood Trihalomethane Concentrations in NHANES 1999–2006

**DOI:** 10.1289/ehp.1306499

**Published:** 2014-03-19

**Authors:** Anne M. Riederer, Radhika Dhingra, Benjamin C. Blount, Kyle Steenland

**Affiliations:** 1 Department of Environmental Health, Rollins School of Public Health, Emory University, Atlanta, Georgia, USA; 2 Tobacco and Volatile Organic Compounds Branch, Division of Laboratory Sciences, National Center for Environmental Health, Centers for Disease Control and Prevention, Atlanta, Georgia, USA

## Abstract

Background: Trihalomethanes (THMs) are water disinfection by-products that have been associated with bladder cancer and adverse birth outcomes. Four THMs (bromoform, chloroform, bromodichloromethane, dibromochloromethane) were measured in blood and tap water of U.S. adults in the National Health and Nutrition Examination Survey (NHANES) 1999–2006. THMs are metabolized to potentially toxic/mutagenic intermediates by cytochrome p450 (CYP) 2D6 and CYP2E1 enzymes.

Objectives: We conducted exploratory analyses of blood THMs, including factors affecting CYP2D6 and CYP2E1 activity.

Methods: We used weighted multivariable regressions to evaluate associations between blood THMs and water concentrations, survey year, and other factors potentially affecting THM exposure or metabolism (e.g., prescription medications, cruciferous vegetables, diabetes, fasting, pregnancy, swimming).

Results: From 1999 to 2006, geometric mean blood and water THM levels dropped in parallel, with decreases of 32%–76% in blood and 38%–52% in water, likely resulting, in part, from the lowering of the total THM drinking water standard in 2002–2004. The strongest predictors of blood THM levels were survey year and water concentration (*n* = 4,232 total THM; *n* = 4,080 bromoform; *n* = 4,582 chloroform; *n* = 4,374 bromodichloromethane; *n* = 4,464 dibromochloromethane). We detected statistically significant inverse associations with diabetes and eating cruciferous vegetables in all but the bromoform model. Medications did not consistently predict blood levels. Afternoon/evening blood samples had lower THM concentrations than morning samples. In a subsample (*n* = 230), air chloroform better predicted blood chloroform than water chloroform, suggesting showering/bathing was a more important source than drinking.

Conclusions: We identified several factors associated with blood THMs that may affect their metabolism. The potential health implications require further study.

Citation: Riederer AM, Dhingra R, Blount BC, Steenland K. 2014. Predictors of blood trihalomethane concentrations in NHANES 1999–2006. Environ Health Perspect 122:695–702; http://dx.doi.org/10.1289/ehp.1306499

## Introduction

Trihalomethanes (THMs) are formed during drinking-water disinfection as by-products of the reaction of chlorine/chloride with organic material and with bromide and iodide in source waters. The U.S. Environmental Protection Agency (EPA) regulates total THM (TTHM)—the sum of chloroform, bromoform, bromodichloromethane (BDCM), and dibromochloromethane (DBCM)—in public drinking water to 0.08 mg/L to reduce potential cancer and reproductive/developmental health risks (U.S. EPA 2001a). The U.S. EPA classifies chloroform as a probable human carcinogen based on animal evidence that ingestion or inhalation at cytotoxic doses produces, via short-lived, toxic intermediates, hepatic and renal neoplasia (U.S. EPA 2001b), even though chloroform is not directly mutagenic or genotoxic (Richardson et al. 2007). *In vitro* studies show that brominated THMs (bromoform, BDCM, DBCM) are activated to mutagenic intermediates by glutathione *S*-transferase-theta-1 (GSTT1) (Cantor et al. 2010; Richardson et al. 2007; Villanueva et al. 2004). The U.S. EPA classifies bromoform and BDCM as probable human carcinogens and DBCM as a possible human carcinogen (U.S. EPA 1991, 1992, 1993). Epidemiologic studies have reported associations between THM exposure and bladder cancer (Villanueva et al. 2004, 2007). There is compelling evidence of associations between THM exposure and preterm delivery and small for gestational age/intrauterine growth restriction, but evidence for other reproductive/developmental outcomes remains inconsistent (Colman et al. 2011; Nieuwenhuijsen et al. 2009).

Because of their complex chemistry and other factors, THM exposure assessment is challenging and has been a key weakness in epidemiologic studies (Blount et al. 2011). Blood levels are a common measure of exposure; typically, the less-toxic parent compounds, not their short-lived toxic/mutagenic metabolites, are used (Blount et al. 2011). Blood THMs decrease within minutes to hours after exposure; however, slower partitioning out of adipose tissue and the relatively high (e.g., daily) frequency of exposure events such as showering/bathing are thought to produce steady-state blood concentrations (Blount et al. 2011). A single blood sample provides a window into this steady-state level.

A number of factors affect blood THM levels. Swimming in chlorinated pools and/or spending time at indoor pools is positively associated with blood THM concentrations (Aggazzotti et al. 1998; Caro and Gallego 2007; Kogevinas et al. 2010). In small-scale studies of U.S. adults, showering/bathing, washing dishes by hand, and ingestion of hot beverages made with tap water are associated with higher blood THMs, with showering/bathing the strongest predictor (Ashley et al. 2005; Backer et al. 2008; Lynberg et al. 2001; Nuckols et al. 2005). In one study (Backer et al. 2008), higher body mass index (BMI) predicted lower postshower blood levels for all THMs except chloroform, and smaller pre-/postshower differences for all THMs. In models controlling for BMI, swimming/sauna activity, dry cleaner visits, hot water intake, and air THMs, Backer et al. (2008) found that *GSTT1*-null (inactive enzyme) participants had higher postshower blood chloroform than *GSTT1*-positive participants.

Chloroform is oxidized in the liver, kidney, and nasal mucosa to trichloromethanol, which degrades to phosgene, which forms cytotoxic adducts (U.S. EPA 2001b). In humans and rats, cytochrome p450 (CYP) 2E1 is the primary enzyme catalyzing this at low chloroform concentrations, such as those after tap-water exposures (Gemma et al. 2003). Treatment of rat liver microsomes with CYP2E1 inducers such as acetone increases chloroform metabolism (Testai et al. 1996). Gemma et al. (2003) observed reduced metabolism of low-concentration chloroform in human liver preparations treated with a CYP2E1 inhibitor. CYP2E1 also metabolizes low-concentration BDCM in human liver preparations (Zhao and Allis 2002). CYP2D6 is active at low BDCM concentrations but with lower catalytic efficiency (Allis and Zhao 2002). Although human data on other brominated THMs are limited, CYP2E1 metabolizes DBCM in rat liver (Pankow et al. 1997). In contrast to GSTT1, which activates the brominated THMs to mutagens, CYP2E1 converts approximately 70–80% of BDCM (and presumably the other brominated THMs) to carbon dioxide via phosgene hydrolysis (summarized by Leavens et al. 2007).

Gene polymorphisms may be important. Backer et al. (2008) found that blood CYP2E1 activity did not predict blood THM levels, but that study subjects with *CYP2D6* polymorphisms indicating decreased activity had higher postshower blood chloroform and BDCM and higher postshower changes in BDCM and DBCM versus baseline.

In adults, *CYP2E1* expression is influenced by age, obesity, diabetes and other chronic diseases, fasting, diet, and exposure to CYP2E1 inducers (e.g., ethanol, benzene, acetone) and substrates (e.g., caffeine, acetaminophen) (Brill et al. 2012; Miksys and Tyndale 2004; Pohl and Scinicariello 2011). Expression is also influenced by ingesting garlic, red peppers, cruciferous vegetables, green/black tea, and watercress, which has been shown to inhibit CYP2E1 after a single ingestion (Neafsey et al. 2009). Cruciferous vegetables also induce the human glutathione *S*-transferase (GST) isozymes GST-α and GST-π (Nijhoff et al. 1995). The relative influence of each factor is not well understood, but overall human interindividual variability in CYP2E1 activity is thought to range from 4- to 20-fold (Neafsey et al. 2009).

THMs were measured in blood and tap water in the 1999–2006 U.S. National Health and Nutrition Examination Survey (NHANES). We used multivariable regression and the NHANES data to explore blood THMs in relation to prescription medications (our primary research interest) and other factors potentially influencing THM metabolism, controlling for tap-water concentrations. Characterizing toxicokinetic and other environmental factors that influence blood THMs will help in understanding how exposures translate into blood concentrations and may provide further justification for using these biomarkers in epidemiologic studies. It may also potentially reduce bias in THM epidemiologic studies by identifying potential confounders and effect modifiers of exposure, even in studies without blood THM data.

## Methods

*NHANES data collection*. NHANES participants provided informed consent, thus we had a waiver from Emory University’s institutional review board for the present analyses. Detailed data collection methods are available at the NHANES website [Centers for Disease Control and Prevention (CDC) 2001]. Briefly, a random subsample (one-fourth in 1999, one-third in 2000–2002 and one-half in 2003–2006) of participants (20–59 years of age in 1999–2004, 12–85 years of age in 2005–2006) was recruited to participate in the THM study during the NHANES medical examination. A venous blood sample was collected during the examination and participants were asked to collect a cold tap-water sample from a bathtub or outside faucet. Home examiners collected samples from participants who could not return theirs within 46–76 hr. Although water samples were collected 2–3 days after blood sampling, we considered them representative of water TTHM concentrations prior to blood sampling, based on evidence that 24-hr average tap-water concentrations remain constant within a season despite hourly fluctuations due to temperature, residence time, and other factors (Chaib and Moschandreas 2008). In NHANES 1999–2000, a randomly selected subset of participants was recruited to wear passive badges (3M™ Organic Vapor Monitor 3520; 3M Corporation, St. Paul, MN) for 48–72 hr after the examination to measure personal air chloroform concentrations.

Badge samples were analyzed using gas chromatography/high resolution mass spectrometry (GC/MS) at contract laboratories, with detection limits varying by badge-wearing duration [typical limit of detection (LOD) of approximately 0.55 μg/m^3^; CDC 2005a]. Whole blood and water samples were analyzed for bromoform, chloroform, BDCM, and DBCM at the National Center for Environmental Health, CDC (Atlanta, GA) using capillary GC/MS. LODs ranged from 0.2 to 2.4 pg/mL for blood and from 0.05 to 0.9 ng/mL for water (CDC 2011a, 2011b). A small percentage of null values (primarily blood chloroform) were reported for results not passing laboratory quality review.

*Imputation of observations below the LOD*. In NHANES 1999–2006, the proportion of above-LOD observations ranged from 48.8% (bromoform) to 95.0% (chloroform) for blood, and 54.0% (bromoform) to 82.7% (chloroform) for water. Following Finkelstein and Verma (2001), we used maximum likelihood estimation (MLE) to impute THM concentrations for below-LOD observations (i.e., nondetects). For each nondetect, we replaced the CDC default LOD/_√_^–^2 value with a randomly selected, below-LOD concentration from the MLE-estimated log-normal distribution for that analyte/medium. For comparison, we calculated separate descriptive statistics for the blood and water measurements using LOD/_√_^–^2 for the nondetects. We added concentrations of the four THMs to get TTHM concentrations, using imputed concentrations of the individual THMs for nondetects. If one or more individual THM concentrations was missing, we considered the TTHM observation missing. Data were missing for approximately 20% of blood samples mainly because of sample loss resulting from spoilage during storage in the laboratory during 1999–2002.

The 1999–2006 data contained one blood bromoform, five blood chloroform, two water bromoform, and one water chloroform concentration exceeding the upper calibration ranges; we considered these to be detects and used the concentrations as reported.

*Covariate coding*. In addition to water, we considered other predictors of blood THMs based on a literature review, including selected *a*) demographic characteristics {age (12 ≤ age < 40, 40 ≤ age ≤ 85 years); highest education level (≤ high school, ≥ college); marital status, which Rivera-Núñez et al. (2012) found strongly predicted blood TTHMs in their study of postpartum women in three U.S. cities [married/not married; following Rivera-Núñez et al. (2012)]; race/ethnicity}; *b*) exposure factors [vigorous or moderate swimming, one or more times in the previous 30 days (yes, no)], and; *c*) toxicokinetic influences [BMI (18.5 > BMI, 18.5 ≤ BMI < 25.0, 25.0 ≤ BMI < 30.0, BMI ≥ 30.0 kg/m^2^)]; pregnancy status (male, female/not pregnant, female/pregnant, missing/female/cannot ascertain); any alcohol, caffeine, garlic, raw cruciferous vegetables, green/black tea, and/or watercress consumed in the previous 24 hr (yes, no); total grams fat consumed the previous 24 hr; smoking (active smoker, nonsmoker/former smoker); diabetes (yes, no); fasting. For race/ethnicity, we created a simple variable indicating whether or not a participant was from the major NHANES race/ethnicity category (non-Hispanic white). Participants were requested to fast overnight before the NHANES examination (CDC 2005b); the median fast length in the THM subsample was 10 hr, so we categorized participants as fasting ≤ 10 hr or > 10 hr. They were also asked whether or not they drank coffee or tea with cream or sugar the day of the examination, with only 1% reporting they had; tap water and/or black coffee/tea or other drinks made with tap water were not asked about. We also included NHANES examination session (morning, afternoon, or evening) to account for the fact that morning participants may have showered/bathed more recently before the blood draw than those in the other sessions. We had no direct data on showering within the previous 24 hr.

Data on these covariates were obtained from the physical activity (CDC 2007a, 2007b) and smoking (CDC 2008b) questionnaires, the demographic (CDC 2009a) examination (CDC 2009b), and dietary interview files (CDC 2008a); these data were collected the day of blood sampling. “Refused”/“don’t know” responses were < 5% for each questionnaire-based variable; we coded these as missing. The NHANES smoking questionnaires changed during 1999–2006 (see Supplemental Material, “Smoking definitions,” p. 2, for details regarding classification of active smokers versus nonsmokers).

Participants reported their use of prescription medications in the previous month; generic drug names were recorded (CDC 2009c). The 6,924 THM subsample participants reported taking 1,450 different drugs or drug combinations. Less than 1.5% of responses were “unknown,” “refused,” “don’t know,” or unspecified (no generic ingredient named); we coded these as missing. We used online drug interaction databases to determine whether or not each generic ingredient was an inducer, inhibitor, or substrate of CYP2D6 or CYP2E1, starting with the SuperCYP database (Preissner et al. 2010), followed by GenomeQuest Inc. (2010), Kanehisa Laboratories (2012) and Flockhart (2007). We created six dichotomous variables indicating whether or not a participant took one or more prescription CYP2D6 or CYP2E1 inducers, inhibitors, and/or substrates, respectively, in the previous month. Some medications were classified as both or as all three; this occurred with approximately 8% of the drugs/drug combinations we evaluated. For simplicity, we assumed independence among the six drug variables, even though some drugs were in more than one category. Because insulin inhibits CYP2E1 according to these databases, it is a potential confounder in the diabetes–blood THM association. However, because < 1% of the subsample reported taking insulin, we did not examine this further. We also recognize that insulin and other medications may lie on the causal pathway of some associations investigated; adjusting for these in multivariable models might have introduced bias.

We did not have complete data on tap-water consumption outside the home. Data available for 2005–2006 showed 19% of participants consuming some tap water (plain drinking water; not food/beverages made with water) outside the home in the 24 hr before the NHANES examination. Unweighted Wilcoxon rank sum tests showed no statistically significant differences in blood THMs between those who did or did not drink tap water outside the home, except for chloroform (*p* ≤ 0.05), which had higher mean levels in the no-tap-water-outside-the-home group. Because we did not have data for other survey years, we could not include this covariate in our models. We did have 1999–2006 data on hot drinks (e.g., coffee, tea, cocoa, instant soup) consumed outside the home in the 24 hr before the examination. We assumed these were made with tap water and included a hot-drinks-consumed-outside-the-home variable (any vs. none) in our model-building efforts.

*Statistical analyses*. We used SUDAAN, version 10.0 (Research Triangle Institute, Research Triangle Park, NC), and SAS, version 9.2 (SAS Institute Inc., Cary, NC), for statistical analyses. To obtain point and range [i.e., 95% confidence intervals (CI)] estimates, we created 8-year sample weights from the THM subsample weights, then used the NHANES survey design variables and 8-year weights to calculate descriptive statistics (CDC 2006). We calculated Pearson correlation coefficients for all possible pairings of THMs in blood and water using natural log-transformed concentrations.

We used weighted linear multivariable regression to evaluate associations between natural log-transformed blood THM levels and the selected covariates, controlling for water concentrations (e.g., controlling for water chloroform in the blood chloroform model). Five models were constructed—one for each individual THM and one for TTHM. Model building consisted of fitting a main effects full model with all 22 selected variables and NHANES survey year, then removing variables with the highest Wald statistic one by one until all remaining had *p*-values ≤ 0.10. We eliminated variables manually because SUDAAN did not have an automated stepwise procedure. The SUDAAN regression procedure excludes observations with missing values for any model variables. We tested age as both a continuous and categorical (12 ≤ age < 40 and 40 ≤ age ≤ 85 years) variable in separate models for each THM, but neither version met our criterion for inclusion in any final models. We forced the six drug ingestion terms in at each step because estimating the effects of prescription medications on blood THMs was an original motivation for our study. We tested two-way interactions between water concentration and other variables in the final model, based on the *a priori* assumption that water concentrations would be the strongest influence on blood levels. Our criterion for including an interaction term in the final model was the same as that for main effects (i.e., Wald *p* ≤ 0.10). We checked correlations between main effects variables in the final models and evaluated model assumptions (normality, homoscedasticity) by examining plots of predicted values versus residuals and normal probability plots of residuals. We examined effects of extreme values, identified visually on box plots of the log-transformed blood data, by fitting the final models with and without these observations and comparing results.

Although the 1999–2000 air chloroform measurements were collected after the blood draw, we considered them a reasonable proxy for typical inhalation exposures. We conducted a subanalysis to evaluate the influence of air versus water chloroform on blood levels, fitting the final chloroform model to the 1999–2000 data both with and without the air measurements and comparing results.

## Results

*Descriptive statistics*. Table 1 shows descriptive statistics for the NHANES 1999–2006 THM subsample (*n* = 6,924); the statistics were similar when the sample was restricted to observations with non-missing blood THM measurements. The majority of participants (82.6%) had a private/public water company as their source of tap water versus 15.7% who had a private/public well (data not shown). Overall, 28.8% used home water treatment devices (e.g., filter, softener, aerator), with 25.9% of those on water-company water and 45.8% of those on well water using them. The most commonly eaten raw cruciferous vegetables (data not shown) were mustard/horseradish (11.7% of participants) and cabbage (e.g., coleslaw; 4.4% of participants). Approximately half the participants took any prescription medication in the previous month (Table 2). Of these, 4.8%, 27.6%, and 26.6%, respectively, took one or more CYP2D6 inducers, inhibitors, or substrates, and 5.1%, 4.2%, and 10.7%, respectively, took one or more CYP2E1 inducers, inhibitors, or substrates.

**Table 1:** Descriptive statistics of the NHANES 1999–2006 THM subsample (*n* = 6,924).

Variable	Unweighted *n* (%)	Weighted % (95% CI)
Age (years)
12 ≤ age < 40	3,894 (56.2)	50.5 (48.5, 52.5)
40 ≤ age ≤ 85	3,030 (43.8)	49.5 (47.5, 51.5)
Pregnancy status
Male	3,266 (47.2)	51.7 (50.2, 53.1)
Female, not pregnant	2,680 (38.7)	46.0 (44.5, 47.4)
Female, pregnant	473 (6.8)	2.4 (2.0, 2.8)
Missing/female/cannot ascertain	505 (7.3)	5.6 (4.6, 6.5)
Highest education level
High school/less	2,952 (50.6)	42.5 (40.1, 44.9)
Some college/higher	2,884 (49.4)	57.5 (55.1, 59.9)
Marital status
Married	3,173 (48.1)	55.1 (52.9, 57.2)
Not married	3,423 (51.9)	45.0 (42.8, 47.1)
BMI (kg/m^2^)
18.5 > BMI	335 (4.8)	3.7 (3.0, 4.3)
18.5 ≤ BMI < 25.0	2,330 (33.7)	34.8 (33.0, 36.5)
25.0 ≤ BMI < 30.0	2,129 (30.8)	31.1 (29.6, 32.6)
BMI ≥ 30.0	2,130 (30.8)	30.5 (28.7, 32.3)
Doctor-diagnosed diabetes
Yes	433 (6.3)	5.3 (4.7, 5.9)
No	6,421 (93.7)	94.6 (94.0, 95.2)
Alcohol in previous 24 hr
Yes	1,537 (23.3)	29.1 (27.5, 30.6)
No	5,050 (76.7)	70.9 (69.4, 72.5)
Caffeine in previous 24 hr
Yes	5,407 (82.1)	87.7 (86.7, 88.7)
No	1,180 (17.9)	12.3 (11.3, 13.3)
Green/black tea in previous 24 hr
Yes	1,256 (19.1)	21.8 (20.2, 23.5)
No	5,331 (80.9)	78.2 (76.5, 79.8)
Hot drinks outside home in previous 24 hr
Yes	1,182 (17.9)	21.8 (19.9, 23.7)
No	5,405 (82.1)	78.3 (76.3, 80.1)
Raw cruciferous vegetables in previous 24 hr
Yes	1,024 (15.6)	18.2 (16.8, 19.8)
No	5,563 (84.5)	81.8 (80.2, 83.2)
Smoking status
Active smoker	1,775 (26.5)	30.0 (27.9, 32.1)
Non­smoker/former smoker	4,936 (73.6)	69.5 (67.3, 71.6)
Food fast before NHANES examination
≤ 10 hr	4,025 (58.1)	56.6 (55.0, 58.3)
> 10 hr	2,899 (41.9)	43.4 (41.7, 45.0)
Examination session
Morning	3,345 (48.3)	48.3 (46.6, 50.0)
Afternoon	2,401 (34.7)	32.8 (31.2, 34.4)
Evening	1,178 (17.0)	18.9 (17.8, 20.0)
Swimming in previous 30 days
Yes	94 (1.4)	1.5 (1.1, 2.0)
No/unable	6,795 (98.6)	98.5 (97.9, 98.9)

**Table 2:** Prescription medication use in the NHANES 1999–2006 THM subsample (*n* = 6,924).

Prescription medication use in previous month	Unweighted *n* (%)	Weighted % (95% CI)
Any medications
Yes	2,973 (42.9)	48.7 (46.7, 50.8)
No	3,941 (56.9)	51.3 (49.2, 53.4)
Missing/refused/don’t know	10 (0.1)	0.14 (0.0, 0.3)
Medications affecting CYP2D6^*a*^
≥ 1 inducers	292 (4.2)	4.8 (4.1, 5.6)
≥ 1 inhibitors	1,678 (24.2)	27.6 (25.9, 29.3)
≥ 1 substrates	1,583 (22.9)	26.6 (25.1, 28.2)
No medications	3,941 (57.0)	51.7 (49.6, 53.7)
Missing/unspecified	62 (0.9)	0.9 (0.6, 1.2)
Medications affecting CYP2E1^*b*^
≥ 1 inducers	316 (4.6)	5.1 (4.4, 5.8)
≥ 1 inhibitors	272 (3.9)	4.2 (3.6, 4.9)
≥ 1 substrates	644 (9.3)	10.6 (9.7, 11.7)
No medications	3,941 (56.9)	51.2 (49.2, 53.3)
Missing/unspecified	66 (1.0)	1.0 (0.7, 1.3)
^***a***^Numbers equal more than the total number of participants because some reported taking multiple medications affecting CYP2D6. ^***b***^Numbers do not equal the total because only participants taking medications that affect CYP2E1 are shown.		

Table 3 presents descriptive statistics for blood and water THMs. For each THM, analytical LODs for blood and water differed significantly by survey year according to nonparametric Kruskal–Wallis tests (*p* < 0.01) (data not shown). Approximately 50% of the water samples in 1999–2000, 67% in 2001–2002, and 80–90% in 2003–2006 had matching blood measurements. Detection frequencies and geometric mean (GM) water concentrations were nearly identical for the matched versus total data (data not shown), thus only the total data are presented.

**Table 3:** Weighted detection frequencies, GMs, medians, and 95% CIs of THM concentrations in blood and water in NHANES 1999–2006.

Sample type/THM	*n*	Missing	Percent > LOD (95% CI)	GM (95% CI)^*a*^	Median (95% CI)^*a*^
Whole blood (pg/mL)
Bromoform	5,430	1,494	48.8 (42.7, 54.8)	0.8 (0.7, 0.9)	0.8 (0.7, 1.0)
Chloroform	5,332	1,592	94.9 (93.0, 96.8)	12.9 (11.8, 14.0)	12.9 (11.7, 14.0)
BDCM	5,600	1,324	79.1 (74.8, 83.5)	1.5 (1.3, 1.7)	1.6 (1.3, 1.8)
DBCM	5,586	1,338	56.3 (50.4, 62.2)	0.6 (0.5, 0.7)	0.6 (0.5, 0.8)
TTHM>	4,982	1,942	34.3 (29.8, 39.1)	18.7 (17.3, 20.1)	18.1 (16.5, 20.0)
Tap water (ng/mL)
Bromoform	6,233	691	54.0 (48.1, 60.0)	0.1 (0.1, 0.2)	0.1 (0.1, 0.2)
Chloroform	6,234	690	82.7 (79.6, 85.9)	4.2 (3.3, 5.4)	12.0 (9.1, 15.1)
BDCM	6,228	696	82.5 (79.1, 86.0)	1.8 (1.4, 2.2)	4.5 (3.6, 5.6)
DBCM	6,246	678	79.5 (75.6, 83.4)	0.9 (0.7, 1.1)	1.1 (0.8, 1.6)
TTHM	6,183	741	50.3 (44.6, 55.9)	10.1 (8.2, 12.2)	25.3 (20.7, 29.2)
TTHM, sum of bromoform, chloroform, BDCM, DBCM; percent > LOD includes only TTHM observations with detectable concentrations of each; GM and median include imputed < LOD observations. ^***a***^Values < LOD imputed following Finkelstein and Verma (2001); LODs varied by survey year.					

**Figure 1:**
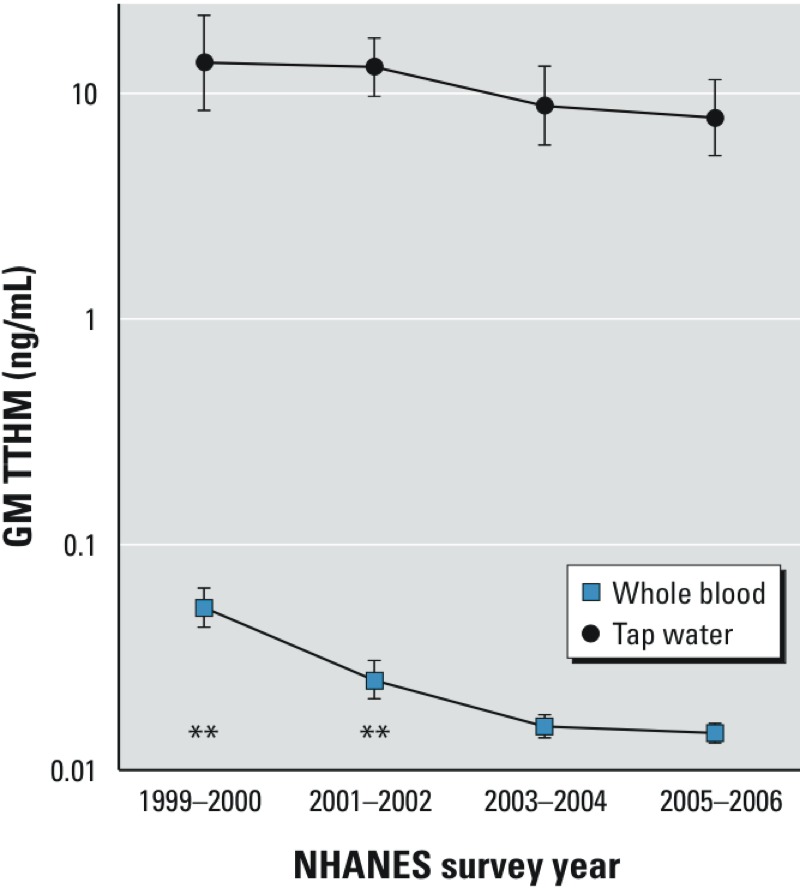
GM (unadjusted) whole-blood and tap-water TTHM (ng/mL) by NHANES survey year, 1999–2006 (error bars represent 95% CIs calculated using NHANES weights and survey design variables). **p < 0.01; log blood TTHM was significantly higher in 1999–2000 and 2001–2002 compared with base year 2005–2006 in multiple regression analysis adjusting for log water TTHM, diabetes status, cruciferous vegetable consumption, NHANES examination session, and recent use of prescription medications affecting CYP2D6 and CYP2E1 enzymes.

In general, GM blood and water THMs decreased during 1999–2006, regardless of imputation strategy used for the nondetects (i.e., MLE vs. LOD/_√_^–^2). Figure 1 shows declining TTHM levels in blood and water during this period, and Supplemental Material Tables S1 and S2 show THM detection frequencies and GMs by survey year for all observations with available measurements. Approximately 8%, 2%, 3%, and 6% (data not shown) of NHANES 1999–2000, 2001–2002, 2003–2004, and 2005–2006 participants, respectively, had TTHM tap-water concentrations exceeding the U.S. EPA’s 0.08 mg/L standard (U.S. EPA 2001a).

All correlation coefficients (ρ) were statistically significant at the α = 0.05 level, except for chloroform and bromoform in water (*p* = 0.08), with the largest between TTHM and chloroform in blood (ρ = 0.91) and TTHM and chloroform and TTHM and BDCM in water (ρ = 0.94 and ρ = 0.95, respectively), and the smallest between chloroform and bromoform in blood (ρ = 0.04) and water (ρ = 0.02) (see Supplemental Material, Table S3).

*Regression analyses*. Table 4 shows results of the multivariable regression analyses. The final models explained 34%, 22%, 32%, 44%, and 44% of the variance in blood levels of TTHM, bromoform, chloroform, BDCM, and DBCM, respectively. Residual plots showed model assumptions were met (data not shown). Removing several extreme values did not change the regression results. Survey year and water THM concentrations were the strongest predictors, in comparison with the other predictors, of blood THM levels in all five models. For most THMs, the models also showed statistically significant inverse associations between blood levels and diabetes and eating raw cruciferous vegetables. For each significant variable in the final models, univariate regression coefficients were within 0–50% of the corresponding multivariable coefficients, except for cruciferous vegetables, BMI group, and pregnancy status, which were within 50–120% (data not shown). Including water (the main predictor) in bivariate models reduced the differences to 0–65%, with only diabetes showing a change > 30%, suggesting water was the main confounder (data not shown).

**Table 4:** Variables associated with log blood THMs in NHANES 1999–2006 in weighted multiple regression models.^*a*^

THM/variable	β (95% CI)	SE	*p-*Value	Multiple *R*^2^
TTHM (*n *= 4,232)				0.34
Survey year
1999–2000 vs. 2005–2006	1.10 (0.86, 1.34)	0.12	< 0.01
2001–2002 vs. 2005–2006	0.38 (0.21, 0.56)	0.09	< 0.01
2003–2004 vs. 2005–2006	0.04 (–0.09, 0.18)	0.07	0.54
Log water TTHM	0.20 (0.17, 0.22)	0.01	< 0.01
Diabetes	–0.14 (–0.25, –0.02)	0.06	0.02
Cruciferous vegetables	–0.12 (–0.21, –0.02)	0.05	0.01
Examination session
Evening vs. morning	–0.16 (–0.29, –0.03)	0.06	0.02
Afternoon vs. morning	–0.08 (–0.16, 0.00)	0.04	0.05
CYP2D6
Inducer(s)	0.27 (–0.09, 0.64)	0.18	0.14
Inhibitor(s)	–0.05 (–0.16, 0.05)	0.05	0.32
Substrate(s)	0.07 (–0.05, 0.19)	0.06	0.26
CYP2E1
Inducer(s)	–0.15 (–0.54, 0.24)	0.20	0.45
Inhibitor(s)	0.01 (–0.18, 0.20)	0.10	0.92
Substrate(s)	–0.00 (–0.15, 0.15)	0.08	1.00
Chloroform (*n *= 4,582)				0.32
Survey year
1999–2000 vs. 2005–2006	1.28 (1.03, 1.54)	0.13	< 0.01
2001–2002 vs. 2005–2006	0.38 (0.19, 0.57)	0.09	< 0.01
2003–2004 vs. 2005–2006	0.00 (–0.16, 0.17)	0.08	0.97
Log water chloroform	0.17 (0.14, 0.19)	0.01	< 0.01
Diabetes	–0.15 (–0.28, –0.02)	0.07	0.03
Cruciferous vegetables	–0.08 (–0.18, 0.02)	0.05	0.10
Examination session
Evening vs. morning	–0.14 (–0.28, –0.01)	0.07	0.04
Afternoon vs. morning	–0.12 (–0.20, –0.04)	0.04	0.01
CYP2D6
Inducer(s)	0.30 (–0.10, 0.69)	0.20	0.14
Inhibitor(s)	–0.02 (–0.15, 0.11)	0.07	0.77
Substrate(s)	0.03 (–0.10, 0.17)	0.07	0.64
CYP2E1
Inducer(s)	–0.18 (–0.58, 0.21)	0.20	0.36
Inhibitor(s)	0.06 (–0.16, 0.29)	0.11	0.58
Substrate(s)	0.07 (–0.09, 0.22)	0.08	0.41
Bromoform (*n *= 4,080)				0.22
Survey year
1999–2000 vs. 2005–2006	0.26 (0.07, 0.45)	0.09	0.01
2001–2002 vs. 2005–2006	0.75 (0.41, 1.08)	0.17	< 0.01
2003–2004 vs. 2005–2006	0.35 (0.06, 0.64)	0.14	0.02
Log water bromoform	0.22 (0.19, 0.25)	0.02	< 0.01
CYP2D6
Inducer(s)	0.28 (–0.09, 0.64)	0.18	0.14
Inhibitor(s)	–0.10 (–0.25, 0.04)	0.07	0.17
Substrate(s)	0.13 (–0.02, 0.27)	0.07	0.09
CYP2E1
Inducer(s)	–0.29 (–0.62, 0.04)	0.17	0.08
Inhibitor(s)	–0.07 (–0.29, 0.14)	0.11	0.50
Substrate(s)	–0.21 (–0.42, –0.01)	0.10	0.05
Fasted > 10 hr	–0.14 (–0.25, –0.04)	0.05	0.01
Examination session
Evening vs. morning	0.01 (–0.15, 0.16)	0.08	0.94
Afternoon vs. morning	0.12 (–0.01, 0.26)	0.07	0.08
High school vs. college	–0.10 (–0.19, –0.00)	0.05	0.05
BDCM (*n *= 4,374)				0.44
Survey year
1999–2000 vs. 2005–2006	0.21 (0.00, 0.42)	0.10	0.05
2001–2002 vs. 2005–2006	0.33 (0.08, 0.58)	0.12	0.01	
2003–2004 vs. 2005–2006	0.08 (–0.14, 0.29)	0.11	0.48
Log water BDCM	0.33 (0.30, 0.35)	0.01	< 0.01
Pregnancy status
Female, pregnant vs. male	–0.25 (–0.48, –0.02)	0.11	0.03
Female, not pregnant vs. male	0.08 (0.01, 0.16)	0.04	0.02
BMI (kg/m^2^)
18.5 > BMI vs. BMI ≥ 30.0	–0.01 (–0.20, 0.18)	0.10	0.89
18.5 ≤ BMI < 25.0 vs. BMI ≥ 30.0	0.10 (0.02, 0.18)	0.04	0.01
25.0 ≤ BMI < 30.0 vs. BMI ≥ 30.0	0.08 (–0.01, 0.16)	0.04	0.08
Diabetes	–0.16 (–0.34, 0.01)	0.09	0.07
Alcohol	0.13 (0.05, 0.21)	0.04	< 0.01
Cruciferous vegetables	–0.13 (–0.22, –0.03)	0.05	0.01
Smoker	–0.13 (–0.23, –0.04)	0.05	0.01
Examination session
Evening vs. morning	–0.26 (–0.36, –0.16)	0.05	< 0.01
Afternoon vs. morning	–0.07 (–0.15, 0.01)	0.04	0.11
CYP2D6
Inducer(s)	0.31 (–0.13, 0.74)	0.22	0.17
Inhibitor(s)	–0.05 (–0.16, 0.05)	0.05	0.33
Substrate(s)	0.08 (–0.05, 0.20)	0.06	0.22
CYP2E1
Inducer(s)	–0.30 (–0.70, 0.09)	0.20	0.13
Inducer(s) × log water BDCM	–0.06 (–0.12, 0.01)	0.03	0.07
Inhibitor(s)	0.09 (–0.12, 0.30)	0.11	0.40
Substrate(s)	–0.02 (–0.21, 0.16)	0.09	0.80
DBCM (*n *= 4,464)				0.44
Survey year
1999–2000 vs. 2005–2006	0.40 (0.17, 0.63)	0.11	< 0.01
2001–2002 vs. 2005–2006	0.20 (–0.08, 0.49)	0.14	0.16
2003–2004 vs. 2005–2006	0.01 (–0.18, 0.20)	0.09	0.94
Log water DBCM	0.48 (0.44, 0.52)	0.02	< 0.01
Pregnancy status
Female, pregnant vs. male	–0.35 (–0.65, –0.06)	0.15	0.02
Female, not pregnant vs. male	0.09 (0.01, 0.17)	0.04	0.04
Diabetes	–0.26 (–0.48, –0.05)	0.11	0.02
Cruciferous vegetables	–0.10 (–0.21, –0.00)	0.05	0.05
Examination session
Evening vs. morning	–0.29 (–0.42, –0.16)	0.07	< 0.01
Afternoon vs. morning	–0.06 (–0.18, 0.06)	0.06	0.30
CYP2D6
Inducer(s)	–0.05 (–0.42, 0.31)	0.18	0.77
Inhibitor(s)	–0.10 (–0.24, 0.05)	0.07	0.19
Substrate(s)	0.11 (–0.04, 0.25)	0.07	0.14
CYP2E1
Inducer(s)	–0.14 (–0.51, 0.23)	0.18	0.44
Inhibitor(s)	0.13 (–0.10, 0.35)	0.11	0.26
Substrate(s)	0.03 (–0.17, 0.23)	0.10	0.76
Substrate(s) × log water DBCM	–0.11 (–0.20, –0.02)	0.05	0.02
^***a***^Model building consisted of fitting a full model with all variables, then removing those with Wald *p* > 0.10 one by one until all remaining had *p* ≤ 0.10, forcing in the medication terms at each step; two-way interactions between water concentrations and each remaining variable were also tested.				

Taking different classes of drugs did not have marked effects on blood levels of any THM, although there were borderline significant effects in the anticipated direction for bromoform with respect to taking CYP2E1 inducers (*p* = 0.08) and substrates (*p* = 0.05), the effect of which would be to increase metabolism and lower parent compounds in the blood. A significant interaction (*p* = 0.02) was seen for water DBCM and those taking CYP2E1 substrates, indicating that the positive effect per unit of water DBCM on blood DBCM was diminished by 30% among those taking CYP2E1 substrates. This is consistent with the idea that more substrate would increase metabolism, which might diminish the parent compound, and that this in turn might diminish the positive effect of water concentrations on blood concentrations. However, this might simply be a chance finding, despite the nominally significant *p*-value.

Results from the 1999–2000 air subanalysis (*n* = 230) are shown in Supplemental Material, Table S4. Statistically significant (*p* < 0.01) associations were detected between log air chloroform and blood chloroform. Including the air term improved model fit (multiple *R*^2^ = 0.24 vs. 0.19). Including air chloroform reduced the water chloroform coefficient by about half, and increased its *p*-value from < 0.01 to 0.05, indicating that air chloroform is a stronger predictor of blood chloroform than water chloroform. Because chloroform volatilizes during hot water use, air chloroform is an intermediate on the causal pathway between water and blood when dermal or ingestion exposures (i.e., via water) are considered and should not be included in models of water concentrations as predictors of blood levels. However, if air concentrations were available, they would be the preferred predictors of blood chloroform based on our findings and others’ (Backer et al. 2008).

Detection frequencies and GM blood and water THMs were higher for participants on water-company versus well water (see Supplemental Material, Table S5). We did not include the NHANES water source variable in our models because we assumed that water concentrations would reflect source. However, we conducted subanalyses restricting the TTHM, chloroform, and BDCM models to participants on water-company water (85% of participants) (see Supplemental Material, Table S6). Results did not differ markedly, although inverse associations with diabetes, cruciferous vegetables, afternoon/evening examination session, and being pregnant were stronger in the subanalyses. In the restricted TTHM model, for example, the absolute value of the regression coefficients increased by 30%, 13%, and 14% for the morning examination session, diabetes, and cruciferous vegetables terms, respectively, compared with the unrestricted model.

## Discussion

*Decreasing blood and water THMs in 1999–2006*. The U.S. EPA lowered the TTHM drinking-water standard from 0.10 mg/L to 0.08 mg/L for large surface-water systems on 1 January 2002 and smaller systems on 1 January 2004, anticipating that this would produce a 24% average national reduction in THM levels (U.S. EPA 2001a). In their NHANES 1999–2004 analysis, Lakind et al. (2010) found a significant decline in blood chloroform but not in the other THMs. In our 1999–2006 analyses, GM blood levels dropped 32%, 76%, 34%, 49%, and 72%, while GM water levels dropped 45%, 38%, 52%, 52%, and 43% for bromoform, chloroform, BDCM, DBCM, and TTHM, respectively (see Supplemental Material, Table S1), consistent with the U.S. EPA’s lowering of the standard. After recoding nondetects to the highest LOD/_√_^–^2, the drops were 75%, 29%, and 30%, respectively, for chloroform, BDCM, and DBCM in blood, and 22%, 32%, and 42%, respectively, for chloroform, BDCM, and DBCM in water. In contrast, the bromoform GM increased 5% in blood and 19% in water. Because NHANES is not designed to sample the same water systems across survey years, the drop in water THMs may also be partly due to sampling different systems. Decreasing water THMs did not entirely explain the statistically significant declines in blood THMs in our models, given that survey year remained important even with water in the models. NHANES sampled cold instead of hot tap water, possibly diminishing the predictive power of water concentrations. Water from home hot water heaters can contain higher THMs because increased temperatures further drive residual disinfectant reactions (Dion-Fortier et al. 2009). Thus, variability in cold water THMs is not likely to explain fully blood THM variability, particularly if hot showers/baths are the main exposure source. The 1999–2000 subanalysis showed air to be a stronger predictor of blood chloroform than water, indicating the importance of showering/bathing versus drinking water as an exposure source.

*NHANES examination session and blood THMs*. We generally found lower blood THMs among those in the afternoon and evening versus morning examination sessions, perhaps because those in the later sessions had more time to metabolize the THMs absorbed during their morning showers. Although estimates of blood THM half-lives after showering are not available, one study showed an 8-hr half-life for blood chloroform after exposure to dry-cleaner air (Gordon et al. 1988). Showering data are not available for the 1999–2006 THM subsample. However, our earlier analysis of NHANES 1999–2000 data showed the majority of participants (86%) reported showering at least once in 72 hr after the examination (Riederer et al. 2009). Others estimate a frequency of 1 shower/day for U.S. adults (Wilkes et al. 2005). Thus, it is reasonable to assume that many participants showered the morning of their NHANES exams. This finding illustrates the importance of including examination session in analyses of NHANES blood THM data.

*Toxicodynamic influences*. Our models showed that several factors potentially affecting THM metabolism were significantly associated with blood THMs, controlling for water concentrations. Diabetes was statistically significantly associated with lower blood concentrations of TTHM and all the individual THMs except bromoform, whereas obesity (i.e., BMI ≥ 30.0 kg/m^2^) was associated with lower blood BDCM. Diabetes and obesity are complex, interrelated conditions associated with increased *CYP2E1* mRNA expression and/or activity in humans (Aubert et al. 2011; Neafsey et al. 2009). Obesity is also associated with increased clearance of compounds metabolized by CYP2D6 (Brill et al. 2012). Fasting is known to induce CYP2E1 (Neafsey et al. 2009), although we are at a loss to explain why fasting was a statistically significant predictor of blood bromoform but not blood concentrations of the other THMs. Non-CYP mechanisms, such as those affecting THM absorption and distribution, may also help explain why we observed lower blood THMs in diabetic, obese, and fasting participants. In the study by Backer et al. (2008), BMI significantly predicted lower postshower blood THMs, presumably reflecting uptake into lipid compartments (Batterman et al. 2002). Last, we note that insulin and other medications may lie on the causal pathway of some of the associations we investigated.

We also found statistically significant associations between eating raw cruciferous vegetables and some blood THMs. Cruciferous vegetables contain sulfur-based glucosinolates that are hydrolyzed by plant enzymes (released by chewing/chopping) and gut microflora to isothiocyanates, some which inhibit CYP2E1, others which induce GST-α and GST-π, although we are unaware of evidence that these isozymes conjugate THMs (Higdon et al. 2007; Nakamura and Miyoshi 2010; Nijhoff et al. 1995). In our models, the cruciferous vegetables regression coefficients were negative, possibly implying increased THM conjugation and/or absorption or distributional changes.

Other potential toxicodynamic influences in our models included prescription medications and pregnancy status. Alcohol consumption, smoking, and education were also associated with blood THMs, but only in the bromoform (education) and BDCM (alcohol, smoking) models. In the bromoform model, taking prescription CYP2E1 inducers or substrates was associated with lower blood levels at borderline significance (*p* = 0.08 and *p* = 0.05 respectively), possibly indicating increased bromoform metabolism in people taking these drugs. Taking prescription CYP2D6 substrates was associated with higher blood bromoform (*p* = 0.09), possibly indicating competitive inhibition or similar toxicodynamic mechanism. The prescription CYP2E1 inducer–log water concentration interaction was borderline (*p* = 0.07) significantly associated with lower blood BDCM, and the prescription CYP2E1 substrate–log water concentration interaction was significant for blood DBCM (*p* = 0.02). The direction of these interactions was consistent with the idea that having more inducer or substrate could increase THM metabolism, which in turn may attenuate the positive effect of water THM levels on blood THM levels. Because approximately half the U.S. adult population takes prescription medications, many of which affect CYP2D6 or CYP2E1, their influence on THM toxicodynamics may be worthy of further investigation.

In our models, pregnant women had statistically significantly lower blood BDCM and DBCM than men (approximately 25–30% lower, on average, holding other variables constant), whereas nonpregnant women had significantly higher levels (approximately 8–9% higher, on average, holding other variables constant). Pregnancy involves a number of changes affecting xenobiotic metabolism. Increased plasma volume, changes in blood protein binding, and fat accumulation during the first two trimesters could increase the volume of distribution of many compounds (Anderson 2005), resulting in decreased blood concentrations at a given exposure level. Pregnancy also induces both CYP2E1 and CYP2D6, which increase by approximately 50% in the third trimester (Anderson 2005; Choi et al. 2013). However, it remains unclear why nonpregnant women had higher blood BDCM and DBCM than men in our models.

*Limitations*. A major limitation is the cross-sectional nature of the NHANES data, which limits our ability to identify the factors driving the observed associations. In addition, our models explained only 22–44% of the variability in blood THMs, thus other factors appear to be important. We lacked detailed data on factors likely to be important to blood levels. Including personal air concentrations in our models, particularly if measurements were taken the morning of the blood draw, would have improved their explanatory power. More detailed data on frequency and duration of showering, and time since showering at the time of blood sampling, would have been useful. We had no detailed data on possible occupational THM exposures, or blood sample dates if there was seasonal variation. We also lacked data on swimming activity close to the blood draw. Such variables could have confounded the relationships we observed.

Other limitations include the dichotomization of the medication terms, which did not allow us to evaluate the possible influence of taking, for example, multiple CYP2E1 inhibitors, or taking higher versus lower doses; NHANES does not include dose information. Summary scores of exposures to inducers and inhibitors may help explain blood THMs better than dichotomous classifications. Other than a toxicokinetic study of BDCM (Leavens et al. 2007), we are not aware of studies on brominated THM metabolism in humans. However, in addition to CYP2E1, CYP2D6, and GSTT1, other enzymes might be important. Another limitation is potential misclassification in our medication coding. The drug databases we used are based on published literature and thus are works in progress. It is possible, for example, that CYP2D6 or CYP2E1 data were lacking for some of the less commonly used medications and that we mistakenly coded these as having no effect. Another limitation is that for many covariates, including medication and dietary intakes, we had only one-time measurements, which can lead to additional measurement error and may have limited our ability to detect associations.

A final caveat concerns the use of blood THM measurements. Although one-time blood concentrations are thought to reflect steady-state levels in humans (Blount et al. 2011), they may be dominated by peak exposure events (e.g., showering/bathing, swimming) close in time to the blood draw. If peak events occur every few days instead of daily, then multiple days of blood measurements may be needed to provide a more accurate measure of steady-state levels. Use of longer-lived exposure markers, such as protein or DNA adducts (Blount et al. 2011), might help avoid this limitation.

## Conclusions

Survey year and water THMs were the strongest predictors of blood THMs in our analyses. We also found statistically significant associations between blood THMs and potential toxicodynamic influences, controlling for water levels. Inverse associations may indicate that these factors alter THM metabolism, although additional research is needed to determine whether changes in THM absorption and distribution also play a role and to evaluate the potential health implications of these findings.

## Supplemental Material

(365 KB) PDF
