# Supplementary material for: Predictors of Blood Trihalomethane Concentrations in NHANES 1999–2006
Source: Environ Health Perspect. 2014 Mar 19;122(7):695–702. doi: 10.1289/ehp.1306499 (PMC4080535; doi:10.1289/ehp.1306499)
Supplement: (365 KB) PDF [file ehp.1306499.s001.pdf]

Environmental Health Perspectives doi:10.1289/ehp.1306499

Supplemental Material

Predictors of Blood Trihalomethane Concentrations in NHANES 1999–2006

Anne M. Riederer, Radhika Dhingra, Benjamin C. Blount, and Kyle Steenland

Table of Contents **Page**

Smoking definitions **2**

References **3**

Table S1. Weighted detection frequencies and geometric means of blood and water THM concentrations by NHANES survey year, 1999–2002—all observations with available data. **4**

Table S2. Weighted detection frequencies and geometric means of blood and water THM concentrations by NHANES survey year, 2003–2006 – all observations with available data. **5**

Table S3. Pearson correlation coefficients (unweighted) between THMs in blood and tap water, NHANES 1999–2006. **6**

Table S4. Variables associated with log blood chloroform in NHANES 1999–2000 in weighted multiple regression models, including and not including personal air chloroform concentrations. **7**

Table S5. Weighted detection frequencies, geometric means, medians, and 95% confidence intervals of THM concentrations in whole blood and tap water in NHANES 1999, 2006, by home tap water source. **9**

Table S6. Sub-analyses of TTHM, chloroform and BDCM models restricted to participants on private/public water company water. **10**

Smoking definitions

The NHANES smoking questionnaires changed during 1999-2006. For 1999-2004, we defined active smokers as those with serum cotinine >10 ng/mL (following Tellez-Plaza et al. 2002) or answering yes to “Do you now smoke cigarettes?” or everyday/some days to “Do you now smoke a pipe?” or “Do you now smoke a cigar/cigars?” For 2005-2006, smokers were those with cotinine >10 ng/mL, or answering everyday/some days to “Do you now smoke cigarettes?” or 1-5 days to “During the past 5 days…on how many days did you smoke a pipe/cigar?” or ≥1 to “During the past 5 days…how many pipes/cigars did you smoke each day?” In NHANES 1999-2004, non-/former smokers were those who answered no to questions on whether or not they had smoked ≥100 cigarettes or a pipe/cigar ≥20 times, or yes to those questions but “Not at all” to the questions “Do you now smoke cigarettes/pipe/cigar?” In NHANES 2005-2006, non-/former smokers answered no to the 100 cigarettes question (there were no pipe/cigar questions in 2005-2006), or yes to this question and “Not at all” to “Do you now smoke cigarettes?”

References

Tellez-Plaza M, Navas-Acien A, Caldwell KL, Menke A, Munter P, Guallar E. 2002. Reduction in cadmium exposure in the United States population, 1988-2008: The contribution of declining smoking rates. Environ Health Perspect 120:204-20.

Table S1. Weighted detection frequencies and geometric means of blood and water THM concentrations by NHANES survey year, 1999-2002—all observations with available data.

Medium/THM	99-00: N	99-00: % >LOD[a] (95% CI)	99-00: GM[a] (95% CI)	01-02: N	01-02: % >LOD[a] (95% CI)	01-02: GM[a] (95% CI)
**Blood (pg/mL)**						
bromoform	330	74.4 (69.4, 79.3)	0.8 (0.7, 1.0)	774	82.5 (74.0, 91.2)	1.4 (1.0, 2.1)
chloroform	255	100.0 (100.0, 100.0)	41.7 (32.3, 53.8)	744	97.3 (95.8, 98.7)	16.5 (13.4, 20.3)
BDCM	354	96.6 (94.4, 98.9)	1.9 (1.6, 2.3)	785	98.2 (97.4, 98.9)	2.2 (1.8, 2.7)
DBCM	350	87.2 (81.5, 92.8)	1.0 (0.8, 1.3)	781	76.0 (68.7, 83.3)	0.8 (0.5, 1.1)
TTHM[b]	234	75.2 (68.3, 81.0)	52.5 (43.0, 64.1)	664	61.5 (45.3, 75.5)	25.0 (20.7, 30.6)
**Water (ng/mL)**						
bromoform	617	68.7 (61.1, 76.4)	0.2 (0.1, 0.3)	1,038	57.0 (45.1, 68.9)	0.1 (0.1, 0.2)
chloroform	630	84.0 (77.0, 91.0)	5.6 (3.0, 10.7)	1,038	85.9 (81.8, 90.0)	5.5 (3.6, 8.6)
BDCM	622	83.1 (76.6, 89.7)	2.8 (1.8, 4.3)	1,024	87.6 (84.4, 90.7)	2.3 (1.7, 3.1)
DBCM	617	84.0 (78.4, 89.7)	1.3 (1.0, 1.9)	1,047	83.6 (77.5, 89.6)	0.9 (0.6, 1.5)
TTHM[b]	614	63.1 (55.2, 70.4)	13.7 (8.4, 22.2)	1,008	54.1 (43.5, 64.3)	13.1 (9.7, 17.6)

LOD, limit of detection. CI, confidence interval. GM, geometric mean. BDCM, bromodichloromethane. DBCM, dibromochloromethane.

[a]GMs and CIs calculated using imputed values for <LOD observations. [b]TTHM = sum of bromoform, chloroform, BDCM, and DBCM; % >LOD includes only TTHM observations with detectable concentrations of each; geometric means include imputed values for <LOD observations.

Table S2. Weighted detection frequencies and geometric means of blood and water THM concentrations by NHANES survey year, 2003-2006 – all observations with available data.

Medium/THM	03-04: N	03-04: % >LOD[a] (95% CI)	03-04: GM[a] (95% CI)	05-06: N	05-06: % >LOD[a] (95% CI)	05-06: GM[a] (95% CI)
**Blood (pg/mL)**						
bromoform	1,310	40.3 (22.7, 57.8)	0.9 (0.6, 1.2)	3,016	30.8 (23.4, 38.2)	0.6 (0.5, 0.7)
chloroform	1,222	93.4 (89.7, 97.1)	10.2 (8.5, 12.1)	3,111	93.9 (89.9, 97.9)	10.1 (9.0, 11.3)
BDCM	1,322	73.4 (65.9, 80.9)	1.4 (1.1, 1.8)	3,139	68.4(58.8, 77.9)	1.2 (0.9, 1.7)
DBCM	1,333	47.7 (38.6, 56.9)	0.5 (0.4, 0.7)	3,122	42.7 (30.4, 55.1)	0.5 (0.4, 0.7)
TTHM[b]	1,199	22.1 (16.0, 29.6)	15.6 (13.9, 17.6)	2,885	21.9 (16.2, 28.8)	14.6 (13.2, 16.1)
**Water (ng/mL)**						
bromoform	1,255	52.0 (37.9, 66.0)	0.1 (0.1, 0.2)	3,323	44.5 (32.2, 56.9)	0.1 (0.1, 0.2)
chloroform	1,240	81.5 (72.7, 90.2)	3.2 (1.7, 6.1)	3,326	81.0 (75.0, 87.0)	3.5 (2.2, 5.6)
BDCM	1,255	83.9 (75.8, 91.9)	1.4 (0.9, 2.3)	3,327	78.6 (70.8, 86.3)	1.3 (0.8, 2.3)
DBCM	1,255	80.9 (71.3, 90.5)	0.8 (0.5, 1.2)	3,327	73.6 (65.0, 82.2)	0.7 (0.4, 1.1)
TTHM[b]	1,240	46.1 (34.7, 57.8)	8.8 (5.9, 13.2)	3,321	42.9 (32.2, 54.3)	7.8 (5.3, 11.5)

LOD, limit of detection. CI, confidence interval. GM, geometric mean. BDCM, bromodichloromethane. DBCM, dibromochloromethane.

[a]GMs and CIs calculated using imputed values for <LOD observations. [b]TTHM = sum of bromoform, chloroform, BDCM, and DBCM; % >LOD includes only TTHM observations with detectable concentrations of each; geometric means include imputed values for <LOD observation.

Table S3. Pearson correlation coefficients (unweighted) between THMs in blood and tap water, NHANES 1999-2006.

Medium/THM	log TTHM	log bromoform	log chloroform	log BDCM
**Whole blood**				
**log bromoform**	0.30; *p*<0.0001 (n=4,982)			
**log chloroform**	0.91; *p*<0.0001 (n=4,982)	0.04; *p*=0.0039 (n=5,116)		
**log BDCM**	0.65; *p*<0.0001 (n=4,982)	0.25; *p*<0.0001 (n=5,383)	0.51; *p*<0.0001 (n=5,297)	
**log DBCM**	0.44; *p*<0.0001 (n=4,982)	0.42; *p*<0.0001 (n=5,368)	0.22; *p*<0.0001 (n=5,271)	0.70; *p*<0.0001 (n=5,538)
**Tap water**				
**log bromoform**	0.28; *p*<0.0001 (n=6,183)			
**log chloroform**	0.94; *p*<0.0001 (n=6,183)	0.02; *p*=0.0831 (n=6,324)		
**log BDCM**	0.95; *p*<0.0001 (n=6,183)	0.34; *p*<0.0001 (n=6,316)	0.88; *p*<0.0001 (n=6,311)	
**log DBCM**	0.72; *p*<0.0001 (n=6,183)	0.69; *p*<0.0001 (n=6,346)	0.54; *p*<0.0001 (n=6,333)	0.82; *p*<0.0001 (n=6,338)

Table S4. Variables associated with log blood chloroform in NHANES 1999-2000 in weighted multiple regression models, including and not including personal air chloroform concentrations.[a]

Model/Variable	β (95% CI)	SE	p-value	Multiple R2
**Including air chloroform (unweighted N=230)**				0.24
log water chloroform	0.07 (0.00, 0.15)	0.04	0.05	
log air chloroform	0.19 (0.08, 0.30)	0.06	<0.01	
CYP2D6				
inducer	-0.26 (-0.72, 0.20)	0.23	0.27	
inhibitor	-0.07 (-0.57, 0.43)	0.25	0.79	
substrate	0.15 (-0.36, 0.65)	0.25	0.57	
CYP2E1				
inducer	-0.08 (-0.83, 0.68)	0.38	0.84	
inhibitor	0.91 (0.13, 1.69)	0.39	0.02	
substrate	0.22 (-0.23, 0.66)	0.22	0.33	
diabetes	-0.55 (-1.02, -0.08)	0.24	0.02	
cruciferous vegetables	-0.31 (-0.58, -0.04)	0.13	0.03	
exam session				
evening vs. morning	-0.34 (-0.64, -0.05)	0.15	0.02	
afternoon vs. morning	-0.16 (-0.41, 0.09)	0.13	0.21	
**Not including air chloroform, but restricted to observations with air measurements (unweighted N=230)**				0.19
log water chloroform	0.13 (-0.81, 0.62)	0.03	<0.01	
CYP2D6				
inducer	0.06 (-0.27, 0.39)	0.17	0.72	
inhibitor	0.03 (-0.49, 0.55)	0.26	0.90	
substrate	0.10 (-0.45, 0.64)	0.27	0.72	
CYP2E1				
inducer	-0.10 (-0.81, 0.62)	0.36	0.79	
inhibitor	1.00 (0.25, 1.74)	0.37	<0.01	
substrate	-0.03 (-0.39, 0.34)	0.18	0.88	
diabetes	-0.49 (-0.90, -0.08)	0.21	0.02	
cruciferous vegetables	-0.31 (-0.57, -0.04)	0.13	0.03	
exam session				
evening vs. morning	-0.45 (-0.79, -0.11)	0.17	0.01	
afternoon vs. morning	-0.26 (-0.52, 0.01)	0.13	0.06	

CI, confidence interval. SE, standard error.

[a]Model building consisted of fitting a full model with all variables then removing those with Wald $p>0.10$ one-by-one until all remaining had $p\leq0.10$, forcing in the drug ingestion terms at each step. Two-way interactions between water concentrations and each remaining variable were also tested. Note that an interaction term between water and air chloroform was tested but not statistically significant.

Table S5. Weighted detection frequencies, geometric means, medians, and 95% confidence intervals of THM concentrations in whole blood and tap water in NHANES 1999, 2006, by home tap water source.

Sample type/THM (units)	% >LOD[a] (95% CI)	Geo. mean[b] (95% CI)	Median[b] (95% CI)
**Whole blood**			
bromoform (pg/mL)			
private/public water co.	53.0 (46.5, 59.5)	0.9 (0.8, 1.0)	0.9 (0.8, 1.0)
private/public well	27.8 (19.6, 37.9)	0.5 (0.4, 0.6)	0.5 (0.4, 0.6)
chloroform (pg/mL)			
private/public water co.	96.8 (95.3, 97.8)	14.6 (13.2, 16.0)	14.0 (12.9, 16.3)
private/public well	84.8 (76.3, 31.5)	7.0 (5.8, 8.4)	6.3 (5.2, 7.7)
BDCM (pg/mL)			
private/public water co.	86.9 (82.9, 90.1)	1.9 (1.6, 2.2)	2.0 (1.8, 2.3)
private/public well	41.4 (33.4, 49.9)	0.5 (0.4–0.6)	0.5 (0.4, 0.5)
DBCM (pg/mL)			
private/public water co.	63.9 (57.6, 69.7)	0.7 (0.6, 0.9)	0.8 (0.6, 0.9)
private/public well	19.8 (14.1, 27.1)	0.2 (0.2, 0.3)	0.2 (0.2, 0.3)
**Tap water**			
bromoform (pg/mL)			
private/public water co.	61.3 (54.8, 67.4)	0.2 (0.1, 0.2)	0.2 (0.1, 0.2)
private/public well	14.0 (10.1, 19.2)	0.0 (0.0, 0.0)	0.0 (0.0, 0.0)
chloroform (pg/mL)			
private/public water co.	93.2 (90.8, 95.1)	8.2 (6.5, 10.3)	16.0 (13.5, 18.9)
private/public well	84.8 (76.3, 90.6)	0.1 (0.1, 0.1)	0.1 (0.1, 0.1)
BDCM (pg/mL)			
private/public water co.	95.0 (93.2, 96.3)	3.5 (2.9, 4.1)	5.6 (4.8, 6.6)
private/public well	15.2 (11.0, 20.5)	0.0 (0.0, 0.1)	0.0 (0.0, 0.0)
DBCM (pg/mL)			
private/public water co.	91.4 (88.3, 93.6)	1.4 (1.1, 1.7)	1.6 (1.2, 2.2)
private/public well	15.2 (11.2, 20.3)	0.1 (0.1, 0.1)	0.1 (0.1, 0.1)

LOD, limit of detection. CI, confidence interval. BDCM, bromodichloromethane. DBCM, dibromochloromethane.

[a]% calculations do not include missing observations. [b]Values below LOD imputed following Finkelstein and Verma (2001); LODs varied by survey year.

Table S6. Sub-analyses of TTHM, chloroform and BDCM models[a] restricted to participants on private/public water company water.

Variable	β (95% CI)	SE	*p*-value	Multiple R^2
**TTHM (N=3,613)**				
survey year				
99-00 vs. 05-06	1.02 (0.78, 1.26)	0.12	<0.01	0.24
01-02 vs. 05-06	0.37 (0.17, 0.57)	0.10	<0.01	
03-04 vs. 05-06	0.05 (-0.09, 0.20)	0.07	0.44	
log water TTHM	0.20 (0.16, 0.24)	0.02	<0.01	
diabetes	-0.16 (-0.30, -0.02)	0.07	0.02	
cruciferous vegetables	-0.14 (-0.25, -0.04)	0.05	0.01	
exam session				
evening vs. morning	-0.23 (-0.35, -0.11)	0.06	<0.01	
afternoon vs. morning	-0.12 (-0.21, -0.04)	0.04	<0.01	
CYP2D6				
inducer	0.36 (0.01, 0.72)	0.18	0.05*	
inhibitor	-0.04 (-0.16, 0.09)	0.06	0.54	
substrate	0.05 (-0.10, 0.19)	0.07	0.52	
CYP2E1				
inducer	-0.24 (-0.62, 0.13)	0.19	0.20	
inhibitor	-0.04 (-0.22, 0.14)	0.09	0.66	
substrate	0.04 (-0.14, 0.23)	0.09	0.62	
**chloroform (N=3,941)**				
survey year				
99-00 vs. 05-06	1.22 (0.96, 1.49)	0.13	<0.01	0.27
01-02 vs. 05-06	0.36 (0.16, 0.56)	0.10	<0.01	
03-04 vs. 05-06	0.01 (-0.16, 0.18)	0.08	0.89	
log water chloroform	0.17 (0.14, 0.20)	0.02	<0.01	
diabetes	-0.19 (-0.34, -0.05)	0.07	0.01	
cruciferous vegetables	-0.13 (-0.25, -0.01)	0.06	0.04	
exam session				
evening vs. morning	-0.22 (-0.35, -0.09)	0.06	<0.01	
afternoon vs. morning	-0.17 (-0.26, -0.08)	0.05	<0.01	
CYP2D6				
inducer	1.18 (0.68, 1.69)	0.25	<0.01*	
inhibitor	0.01 (-0.14, 0.16)	0.07	0.90	
substrate	0.00 (-0.15, 0.16)	0.08	0.95	

Variable	β (95% CI)	SE	*p*-value	Multiple R^2
CYP2E1				
inducer	-1.14 (-1.55, -0.73)	0.21	<0.01*	
inhibitor	0.05 (-0.18, 0.28)	0.11	0.66	
substrate	0.06 (-0.13, 0.25)	0.10	0.53	
log water chloroform*CYP2D6 inducer	-0.31 (-0.50, -0.13)	0.09	<0.01*	
log water chloroform*CYP2E1 inducer	0.39 (0.21, 0.56)	0.09	<0.01*	
**BDCM (N=3,771)**				0.35
survey year				
99-00 vs. 05-06	0.24 (0.01, 0.47)	0.12	0.04	
01-02 vs. 05-06	0.34 (0.07, 0.61)	0.13	0.01	
03-04 vs. 05-06	0.09 (-0.14, 0.32)	0.11	0.43	
log water BDCM	0.34 (0.29, 0.38)	0.02	<0.01	
pregnancy status				
female, pregnant vs. male	-0.32 (-0.58, -0.07)	0.13	0.01	
female, not pregnant vs. male	0.07 (-0.01, 0.15)	0.04	0.07	
diabetes	-0.23 (-0.42, -0.05)	0.09	0.02	
alcohol past 24 hr	0.14 (0.05, 0.22)	0.04	<0.01	
cruciferous vegetables	-0.11 (-0.22, 0.01)	0.06	0.09	
active smoker	-0.23 (-0.36, -0.09)	0.07	<0.01	
exam session				
evening vs. morning	-0.31 (-0.43, -0.20)	0.06	<0.01	
afternoon vs. morning	-0.10 (-0.20, -0.01)	0.05	0.04*	
CYP2D6				
inducer	0.61 (0.13, 1.10)	0.24	0.01*	
inhibitor	-0.15 (-0.31, -0.00)	0.08	0.05*	
substrate	0.06 (-0.07, 0.19)	0.07	0.37	
CYP2E1				
inducer	-0.46 (-0.86, -0.06)	0.18	0.02*	
inhibitor	0.13 (-0.12, 0.37)	0.12	0.30	
substrate	-0.04 (-0.26, 0.18)	0.11	0.01*	
log water BDCM*CYP2D6 inducer	-0.13 (-0.23, -0.03)	0.05	<0.01*	
log water BDCM*CYP2D6 inhibitor	0.07 (0.00, 0.14)	0.04	<0.05*	
log water BDCM*active smoker	0.07 (0.00, 0.14)	0.03	<0.04*	

SE, standard error. *, variable significant in restricted model but not model with all participants. [a]Model building consisted of fitting a full model with all variables then removing those with Wald $p>0.10$ one-by-one until all remaining had $p \leq 0.10$, forcing in the drug ingestion terms at each step. Two-way interactions between water concentrations and each remaining variable were also tested.
